# Reasons Why Combination Therapy Should Be the New Standard of Care to Achieve the LDL-Cholesterol Targets: Lipid-lowering combination therapy

**DOI:** 10.1007/s11886-020-01326-w

**Published:** 2020-06-19

**Authors:** Lluís Masana, Daiana Ibarretxe, Núria Plana

**Affiliations:** 1 Vascular Medicine and Metabolism Unit, Research Unit on Lipids and Atherosclerosis, Sant Joan University Hospital, Universitat Rovira i Virgili, IISPV, C Sant Llorenç, 21, 43201 Reus, Spain; 2 Spanish Biomedical Research Centre in Diabetes and Associated Metabolic Disorders (CIBERDEM), Madrid, Spain

**Keywords:** Lipid-lowering combination therapy, Statins, Ezetimibe, PCSK9 inhibitors, Cardiovascular prevention

## Abstract

**Purpose of Review:**

The aim of this report is to review the scientific evidence supporting that lipid lowering therapy (LLT), beyond statins, reduces cardiovascular risk; therefore, treatment strategies based on lipid-lowering drug combination should be implemented.

**Recent Findings:**

A strong scientific body of evidence supports the effect of statins on cardiovascular risk reduction. Recent trials using non-statin LLT, ezetimibe, and PCSK9 inhibitors have provide scientific evidence about their impact on cardiovascular prevention. Current clinical guidelines still recommend using high-intensity statin monotherapy before considering combination therapy.

**Summary:**

The causal effect of LDL-C on atherosclerosis is well established. Moreover, new RCT, meta-analysis, and Mendelian randomization data, support that the main determinant of risk reduction is the absolute LDL reduction regardless of LLT. Accordingly, the “high-intensity statin therapy” concept should be substituted by “high-intensity lipid lowering therapy.” Combination therapy must become the standard of care of hypercholesterolemia treatment.

## Introduction

In the last 35 years, many randomized controlled trials have tested the impact of lipid-lowering drugs on cardiovascular disease (CVD) risk. In the pre-statin era, the Lipid Research Clinics trial [1] showed a benefit associated with a rather moderate cholesterol reduction induced by cholestyramine. The Scandinavian Survival Simvastatin Study (4S) showed for the first time a significant impact of simvastatin on global mortality and changed the approach to cardiovascular prevention forever [2]. Since then, statins have been mandatory in patients at very high cardiovascular risk. Both the Pravastatin or Atorvastatin Evaluation and Infection Therapy–Thrombolysis in Myocardial Infarction (Prove-it) trial and Treating to New targets (TNT) trial reinforced the impact of these potent LDL cholesterol (LDL-C)-lowering drugs on cardiovascular risk reduction, showing that more efficient statin doses, leading to lower LDL-C levels, lead to an incremental risk reduction [3, 4]. In these studies, the patients in the more active intervention groups achieved LDL-C values below 70 mg/dl. Several scientific societies, including the National Cholesterol Education Program Adult Treatment Panel (ATP) [5], updated their guidelines to recommend an LDL-C target of 70 mg/dl for patients at very high cardiovascular risk. Despite the impressive impact of statins on cardiovascular event reduction, the number of patients with recurrent heart attacks remains unsatisfactorily high. This residual risk is in part due to the presence of other risk factors but also to remaining lipid metabolism alterations that are not properly corrected by statins. HDL-raising drugs were considered a possible approach to complement LDL-lowering therapies. However, when fibrates and niacin were studied along with statin treatment, the results were neutral or even negative [6–9], despite a significant impact on HDL. The greatest disappointment came from the CETP inhibitor family. Despite its huge impact on HDL concentrations, more than doubling HDL cholesterol levels, its effect on cardiovascular prevention was negative [10–12]. Although anacetrapib showed a positive effect on clinical outcomes, it was associated with changes in apoB-containing lipoproteins rather than HDL [13•]. These consistent results, showing no benefit of non-statin lipid-modifying drugs when associated with statins, led to a reconsideration of lipid-lowering therapies (LLTs) by some scientific bodies, including the American College of Cardiology and American Heart Association (ACC/AHA) [14]. Based on the null impact of other LLT and the fact that statin randomized controlled trials (RCT) were not designed to obtain an LDL target, they recommended prescribing high-intensity, high-dose statins to patients at high cardiovascular risk, regardless of LDL-C concentration.

## LDL Cholesterol Reduction Drives Cardiovascular Risk Reduction

The abovementioned message is clear and simple and has helped to increase the number of people on an appropriate high-intensity statin treatment; however, it neglects the importance of LDL reduction.

As shown over and over again, according to the meta-analyses performed by the Cholesterol Treatment Trialist Collaboration (CTTC), the main determinant of risk reduction in the statin RCT is the absolute LDL-C reduction [15].

Since 2013, new RCT, meta-analyses and Mendelian randomization studies have reinforcing the impact of LDL lowering on cardiovascular risk reduction beyond statin therapy.

The Improve-it study was the first RCT of a non-statin LDL-lowering drug showing an extension of the statin effect. Ezetimibe reduced cardiovascular events proportionally to LDL cholesterol reduction, according to the CTTC data [16]. The Improve-it results also showed that achieving LDL concentrations below current recommendations produced an incremental benefit without relevant side effects.

Recent data of RCT using proprotein convertase subtilisin/kexin type 9 inhibitors (PCSK9i) have extended this concept. In the Fourier study, evolocumab 140 mg administered subcutaneously every 2 weeks produced a significant 15% and 20% reduction in the primary and secondary objectives, respectively, after 2.2 years in patients with heart, brain or peripheral atherosclerosis disease who were on optimized LLT therapy and LDL-C concentrations just above 70 mg/dl [17••]. The Fourier data reinforce the fact that the main determinant of cardiovascular risk reduction is LDL lowering. On the other hand, they demonstrated that an LDL concentration of 30 mg/dl leads to an incremental risk reduction in a safe manner.

The Odyssey outcomes trial obtained similar data in a different population [18••]. Patients with acute coronary syndrome treated with alirocumab 75 mg/dl every other week, with up-titrations to 150 mg/dl to obtain an LDL between 50 and 25 mg/dl, reduced the number of events by 15% in 2.8 years of therapy. There was a trend towards lower heart and cardiovascular mortality and a significant reduction in total mortality (nominal *p* < 0.026). From both studies, it was possible to identify subgroups of patients benefiting most from these therapies. Patients with LDL above 100 mg/dl in Odyssey or with recent, recurrent or multivessel atherosclerotic disease, and those with peripheral artery disease in the Fourier study [19•, 20•], obtained a greater benefit from therapy.

Both studies confirm LDL lowering as the cornerstone of cardiovascular prevention by lipid modifying therapies.

This concept has been further reinforced by meta-analyses showing that the correlation between LDL lowering and relative risk reduction (RRR) that has been strongly established for statins by CTTC meta-analyses must be extended to any LDL-lowering method, including diet, resins, ezetimibe, PCSK9i or surgery. These therapies produce exactly the same relative risk reduction (RRR) per unit of LDL cholesterol lowered as statins [21].

Mendelian randomization studies analyze the impact of gene variants leading to protein function modifications on human homeostasis. In the lipid field, they have been used to study the impact on cardiovascular events of gene variants affecting proteins involved in lipid metabolism. This method takes advantage of the natural randomization of these gene variants assuming an even distribution of confounding factors. Mendelian randomization studies have explored the impact of variants in the *HMGCoAr*, *NPC1L1*, *LDLR*, and *PCSK9* genes, among others, mimicking the effects of statins, ezetimibe and PCSK9i. Gene variants leading to lower LDL concentrations determine, in a very robust way, fewer cardiovascular events [22••, 23••]. Interestingly, the magnitude of the RRR per unit of LDL cholesterol reduction is identical regardless of the pathophysiological pathway affected, reinforcing the concept that cardiovascular risk reduction depends on LDL lowering independently of the mechanism involved.

The European Society of Cardiology and the European Atherosclerosis Society (ESC/EAS), taking into account this recent information, have issued a new guideline on dyslipidemia management to reduce cardiovascular events [24••]. The guideline focuses on LDL-C lowering to reduce cardiovascular events, defining different LDL targets according the global cardiovascular risk of the patient (Table 1). For patients at very high CV risk, according to Improve-it, Fourier, and Odyssey outcome study results, the LDL-C target is achieving a concentration below 55 mg/dl. Moreover, a reduction of at least 50% from the basal value is recommended.

**Table 1:** Cardiovascular risk categories, recommended LDL-C targets, evidence class and level, and treatment recommendations addressed to increase target attainment

Disease	Clinical conditions	CV risk category	LDL-C target	Class/level of evidence	Lipid-lowering therapy recommended*
Atherosclerotic cardiovascular disease	Coronary heart disease. Ischemic stroke. Peripheral artery disease or Unequivocal image of ASCVD	Very high	< 55 mg/dl and 50% reduction	I,A	Very-high-intensity oral combination therapy (if target not attained add PCSK9 inhibitors)**
Type 2 diabetes	Organ damage or 3 additional risk factors	Very high	< 55 mg/dl and 50% reduction	I,C	Very-high-intensity oral combination therapy
	> 10 years of duration or up to two additional risk factors	High	< 70 mg/dl and 50% reduction	I,A	High-intensity oral combination therapy (if basal LDL-C < 140 mg/dl high-intensity statin monotherapy can also be effective)
	Age < 50 years and duration < 10 years and no additional risk factors	Moderate	< 100 mg/dl	IIa,A	High-intensity statin monotherapy or combination therapy.
Type 1 diabetes	Long duration (> 20 years)	Very high	< 55 mg/dl and 50% reduction	I,C	Very-high-intensity oral combination therapy
	> 10 years of duration or up to two additional risk factors	High	< 70 mg/dl and 50% reduction	I,A	High-intensity oral combination therapy (If basal LDL-C < 140 mg/dl high-intensity statin monotherapy can also be effective)
	Age < 35 years, and duration < 10 years and no additional risk factors	Moderate	< 100 mg/dl	IIa,A	High-intensity statin monotherapy or combination therapy.
Chronic kidney disease	eGFR < 30 ml/min/m^2^	Very high	< 55 mg/dl and 50% reduction	I,C	Very-high-intensity oral combination therapy
	eGFR > 30 < 60 ml/min/m^2^	High	< 70 mg/dl and 50% reduction	I,A	High-intensity oral combination therapy (if basal LDL-C < 140 mg/dl high-intensity statin monotherapy can also be effective)
Familial hypercholesterolemia	ASCVD or 1 additional major risk factor	Very high	< 55 mg/dl and 50% reduction	IIa,C	Very-high-intensity oral combination therapy (if target not attained add PCSK9 inhibitors)***
	Without additional risk factors	High	< 70 mg/dl and 50% reduction	I,A	Very-high-intensity oral combination therapy
Severe single risk factor	LDL-C above 190 mg/dl	High	< 70 mg/dl and 50% reduction	I,A	Very-high-intensity oral combination therapy
	Blood pressure above 180/110	High	< 70 mg/dl and 50% reduction	I,A	High-intensity oral combination therapy (if basal LDL-C < 140 mg/dl high-intensity statin monotherapy can also be effective)
Combination of risk factors	Score ± 10	Very high	< 55 mg/dl and 50% reduction	I,C	Very high Intensity Oral combination therapy
	Score ± 5 < 10	High	< 70 mg/dl and 50% reduction	I,A	High-intensity oral combination therapy (If basal LDL-C < 140 mg/dl high intensity statin monotherapy can also be effective)
	Score ± 1 < 5	Moderate	< 100 mg/dl	IIa,A	High-intensity statin monotherapy or combination therapy.

*Author’s recommendations

**There is Scientific evidence I,A supporting PCSK9 inhibitors therapy in patients at secondary prevention and LDL above 70 mg/dl. ESC/EAS guidelines recommend using them if LDL-C targets (< 55 mg/dl) are not achieved with oral therapy

***There is no evidence about the use of PCSK9 inhibitors in primary prevention. Its use in familial hypercholesterolemia, even in primary prevention, is widely approved because the pathogenesis of the disease

## LDL Is an Aetiological Factor for Atherosclerosis

The abovementioned data indicate that LDL is not just a cardiovascular risk biomarker but an etiological factor of atherosclerosis. Moreover, basic science, epidemiology, genetics, pathology, clinical, and therapy data are all aligned in showing a strong causal association between LDL cholesterol and atherosclerosis.

A task force of the European Atherosclerosis Society (EAS) has reviewed the epidemiological and clinical evidence and, more recently, the pathogenic bases supporting the causal role of LDL [25••, 26••]. The association between LDL and atherosclerosis fulfils all clinical and epidemiological postulates of causality at the highest level of evidence. Additionally, there is an overwhelming amount of information underlining the pathophysiological pathways involving LDL-C as the primary driver of atherogenesis.

As concluded in the EAS review, consistent evidence from numerous and multiple different types of epidemiological, clinical, biological and genetic studies unequivocally establishes that LDL “causes” atherosclerotic cardiovascular disease.

## Moving from High-Intensity Statin Therapy to High-Intensity LDL-Lowering Therapy

The high-intensity statin therapy concept was established by the ACC/AHA 2013 guidelines and is maintained in the current 2019 version [27•]. It is a pragmatic and easy way to recommend intense LLT for people at high CVD risk. It has increased the number of physicians prescribing an appropriate dose of the most efficient statins. However, as already mentioned, the message shifts the focus of therapy from reducing LDL cholesterol to putting everyone on statins. The lipid-lowering intervention must be focused on LDL reduction. People on high-dose statin who do not achieve appropriate LDL levels remain at high risk [28].

By accepting both LDL causality and LDL lowering as the CVD reduction driver, we should move from the “high-intensity statin therapy” to the “high-intensity LDL-lowering therapy” concept, accepting that high-intensity LDL lowering is much more than just increasing the statin dose and potency [29].

## How Low Can We Go with LDL-C Levels?

The use of lipid-lowering combination therapies has permitted the attainment of extremely low LDL-C concentrations causing concern about its safety. In the Improve-it study, with 7 years mean follow-up, more than 5000 patients achieved an LDL-C below 50 mg/dl and about 1000 below 30 mg/dl [30•]. In the Fourier trial, about 3000 patients had and LDL-C below 20 mg/dl. In both studies, these extremely low LDL-C concentrations were associated to less cardiovascular risk without side effects increment [31•].

Data from preestablished and post hoc analyses from the latest RCT have repetitively shown two facts: [1] very low LDL-C levels are associated to even lower cardiovascular events; [2] Very low LDL-C values do not increase the number of adverse effects, including muscle and liver function, neurocognitive derangements, new-onset diabetes, cancer, hemorrhagic stroke, and so on. The pathophysiological bases of extremely low LDL-C levels safety have been recently reviewed [32••].

From the clinical point of view, these data should be interpreted as scientific evidence for safety. In other words, our goal is achieving the therapeutic targets defined according scientific evidence, however, if LDL-C reaches lower levels, we should not be concerned but the contrary.

## Optimizing the Use of Available Lipid-Lowering Therapies

The aim of this article is not reviewing the pharmacological characteristics of all available lipid-lowering drugs. Our interest is giving instructions to implement, in the best possible way, all LDL-C lowering options, including statins, ezetimibe, PCSK9i, and their combinations. We do not include therapies used in special situations, such as lomitapide or mipomersen, because they are outside the scope of this article.

There is an important interindividual variation in LDL-C-lowering efficacy due to genetic characteristics and environmental factors such as diet, physical activity, comorbidities, basal LDL concentration and adherence [33], among others. Therefore, the data provided from now on should be considered a general approach to guide therapies to improve LDL target achievement. According to ACC/AHA guidelines, statins can be classified according to their LDL-C-lowering efficacy in low-moderate intensity (LDL reductions between 30 and less than 50%) and high intensity (LDL reductions above 50%) [14]. We also know that ezetimibe reduces LDL by approximately 20% and PCSK9 inhibitors by approximately 60%, with the exception of low-dose alirocumab, which reduces LDL by 45–50%.

To calculate the efficacy of combination therapies, we must take into account that individual efficacies cannot be merely added together; otherwise, a high-intensity statin plus ezetimibe plus iPCSK9 would produce (50% plus 20% plus 60%) a 130% LDL-C decrease. To calculate the impact on basal LDL-cholesterol values, the theoretical lowering capacity of each drug must take into account the reduction of the other drugs. The total LDL-lowering efficacy of combination therapies can be calculated using the following formula:

$$ \%A+\%B\ \left(1-\%A\right)+\%C\ \left[1-\left(\%A+\%B\ \left(1-\%A\right)\right)\right] $$

where %A is the theoretical LDL-C reduction induced by drug A, %B by drug B, and %C by drug C [34].

**Fig. 1:**
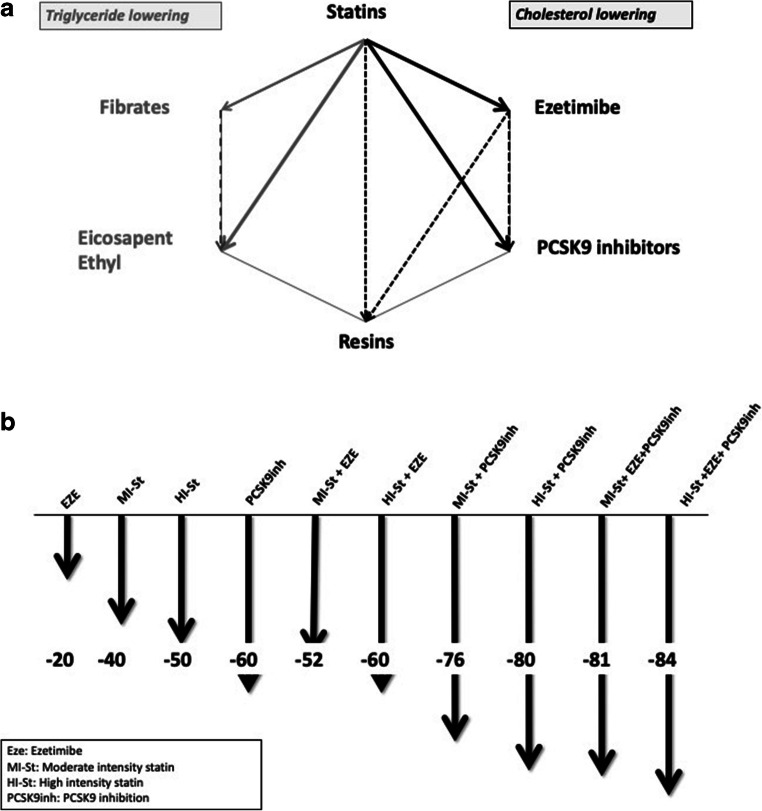
Recommended lipid lowering therapy combinations and its efficacy. **a** Appropriated lipid-lowering combination therapies according scientific evidence. Thicker continuous lines indicate that at least one RCT supports the association. Thinner continuous lines indicate that combination is supported by subgroup analyses. Discontinuous lines indicate that combination potentiates lipid lowering therapy. Triglyceride lowering square indicates that drugs below could be combined with statins in patients with hypertriglyceridemia. Cholesterol-lowering square indicates that drugs below could be combined with statins to reduce LDL-cholesterol. **b** Theoretical percentage reduction on LDL cholesterol concentrations (Fig. 1b created with data from [34])

In Fig. 1, we show the expected theoretical LDL-lowering efficacy of different LLT [31•]. Interestingly, the maximum LDL lowering to be achieved by combined oral therapy (high-intensity statin and ezetimibe) is 60%, similar to PCSK9i. The combination of a moderate-intensity statin and ezetimibe is more efficient than high-intensity statin monotherapy. The maximum LDL-lowering efficacy obtained by high-intensity triple therapy is 84%. Interestingly, the substitution of moderate-intensity statin in this triple therapy produced an 82% reduction (only 2% less that using high-intensity statins). On the other hand, a high-intensity statin plus PCSK9i, without ezetimibe, reduces LDL by 80%, so the contribution of ezetimibe to high-intensity triple therapy is 4%. All these aspects should be taken into consideration when designing lipid-lowering therapies, particularly when prescribing PCSK9i.

PCSK9i are indicated in patients at high risk after high-intensity statin with or without ezetimibe; however, as mentioned above, a moderate-intensity statin plus ezetimibe is more efficient than high-intensity statin monotherapy, and adding ezetimibe to a triple therapy that includes high-intensity statin will contribute very little to the final efficacy. Taking these aspects into consideration will avoid losing time by putting high-risk patients on the most efficient therapy.

Probably, the therapy prior to PCSK9i indication should be just “optimized oral LLT,” meaning a theoretical lipid-lowering efficacy above 50% (monotherapy or combination) and good adherence (above 80%). In our hands, the use of optimized therapy results in a reduction of more than two thirds in patients with indication for PCSK9i [35•].

## Lipid-Lowering Strategies and Useful Drug Combinations

Euroaspire V data pointed out that less than 1 out of 3 patients at very high cardiovascular risk attained the aimed LDL-C levels [36•], and it can be easily anticipated that this proportion will decrease with the new guidelines.

Although the reasons are many, undertreatment must be considered.

Guidelines from Scientific Societies recommend prescribing statins up to the highest tolerated dose and only then considering adding-on non-statin LLT. Based on the abovementioned evidence, we claim using “high-intensity lipid-lowering therapy” tailored to our patient needs, taken into consideration the cardiovascular risk, comorbidities, drug tolerance, and pharmacological efficacy needed to attain the target. In high- and very high-risk patients, apart from attaining ambitious LDL-C targets, at least a 50% reduction is recommended. Considering that high-intensity statin monotherapy provides barely 50% LDL-C reduction, the use of statin monotherapy is almost precluded.

The benefits of using lipid-lowering combination therapies are several. They take advantage of the synergistic effect of drugs acting on different LDL metabolism points: for oral therapies, cholesterol synthesis and absorption, and when using PCSK9i, LDL receptor recycling. The immediate consequence is obtaining similar LDL-lowering efficacy using lower doses; therefore, reducing side effects, such as muscle symptoms or diabetes, that are more prevalent with higher statin doses [37•]. Higher tolerability leads to higher adherence, which can be further reinforced by the use of the available fixed combinations.

Different LLT combinations are shown in Fig. 1. Because more than 30 RCT including approximately 200,000 patients support the scientific evidence of statin benefits, any LLT plan should contain a statin. There is scientific evidence of the beneficial effects of the statin + ezetimibe combination [16] and statin + ezetimibe + PCSK9i [17••, 18••]. The utilization of oral combination therapy is also scientific evidence based. A recent RCT has explored, for the first time, the effect of achieving two different LDL-C targets, regardless of LLT used, on CV events in patients suffering from ischemic stroke. Attaining an LDL-C below 70 mg/dl compared to an LDL between 90 and 110 mg/dl was associated to a 22% relative risk reduction after 3 years follow-up. Interestingly, 40 % of patients in the lower target group were on oral lipid lowering combination therapy compared to 7% in the other group. This study provides scientific evidence about cardiovascular prevention by oral combination therapy in very high-risk patients [38••].

In Table 2, we show different LLT options based in the use of combination therapies to achieve recommended LDL-C objectives.

**Table 2:** LDL-cholesterol-lowering therapies sorted by efficacy categories

	Low-intensity cholesterol-lowering therapy LDL-C < - 30%	Mild-intensity cholesterol-lowering therapy LDL-C > 30% < 50%	High-intensity cholesterol-lowering therapy LDL-C > 50% < 60%	Very-high-intensity cholesterol-lowering therapy LDL-C > 60% < 80%*	Extremely high-intensity cholesterol-lowering therapy LDL-C > 80% < 85%
Oral Monotherapy	Simvastatin 10 Pravastatin 10–20 Lovastatin 10–20 Fluvastatin 40 Pitavastatin 1 Ezetimibe 10	Atorvastatin 10–20 Rosuvastatin 5–10 Simvastatin 20–40 Pravastatin 40 Lovastatin 40 Fluvastatin 80 Pitavastatin 2–4	Atorvastatin 40–80 Rosuvastatin 20–40		
Oral combination therapy		Simvastatin 10 + Ezetimibe 10 Pravastatin 20 + Ezetimibe 10 Lovastatin 20 + Ezetimibe 10 Fluvastatin 40 + Ezetimibe 10 Pitavastatin 1 + Ezetimibe 10	Atorvastatin 10–20 + Ezetimibe 10 Rosuvastatin 5–10 + Ezetimibe 10 Simvastatin 20–40 + Ezetimibe 10 Pravastatin 40 + Ezetimibe 10 Lovastatin 40 + Ezetimibe 10 Fluvastatin 80 + Ezetimibe 10 Pitavastatin 2–4 + Ezetimibe 10	Atorvastatin 40–80 + Ezetimibe 10 Rosuvastatin 20–40 + Ezetimibe 10	
Oral + subcutaneous combination therapy		Alirocumab 75		Alirocumab 150 Evolocumab 140 Atorvastatin 10–20 + Alirocumab/Evolocumab* Rosuvastatin 5–10 + Alirocumab/Evolocumal Simvastatin 40 + Alirocumab/Evolocumab	Atorvastatin 40–80 + Alirocumab/Evolocumab Rosuvastatin 20–40 + Alirocumab/Evolocumab Atorvastatin 40–80 + Ezetimibe 10 + Alirocumab/Evolocumab Rosuvastatin 20–40 + Ezetimibe 10 + Alirocumab/Evolocumab

*Oral combination (high-intensity statin + ezetimibe) and PCSK9 inhibitors in monotherapy produce a reduction in the lower side of the range. Alirocumab 75 mg/15 days in combination provide an LDL-C reduction in the lower side of the range. Alirocumab 150 mg/15 days and Evolocumab 140 mg/15 days provide an LDL reduction in the higher side of the range

Hypertriglyceridemia is a strong CVD risk marker; however, because its causality has not been proven, in patients with hypertriglyceridemia, the main objective of lipid-lowering therapy to lower CVD risk is LDL reduction. Once this objective has been achieved, other therapy actions could be taken into consideration in very high-risk patients. An analysis of an extended follow-up of the Accord trial showed that patients with type 2 diabetes with high triglycerides and low HDL benefitted from fenofibrate add-on statin therapy background [39]. Therefore, cholesterol-lowering therapy combined with fibrates (no gemfibrozil because of severe drug-drug interactions) is an option to treat very high-risk patients with remaining high triglycerides and low HDL after normalizing LDL concentration.

Omega-3 fatty acids (n-3 FA) have been extensively assayed in combination with statins in high-cardiovascular-risk patients. After several neutral-outcome studies [40], the Reduce-it study showed that 4 g a day of icosapent ethyl produced a highly significant impact on cardiovascular events. Therefore, its combination with statins is currently based on scientific evidence [41••].

In the next months, bempedoic acid, an ATP citrate lyase (ACLY) inhibitor, will be clinically available. Its profile is similar to that of ezetimibe, and its combination with ezetimibe and statins will also be clinically useful [42].

## Conclusions

A robust body of scientific evidence supports the impact of LDL reduction on cardiovascular prevention. LDL cholesterol is a causal factor of atherosclerosis. On the other hand, the preventive effect of lipid-lowering drugs is due to their LDL-lowering effect, according to RCT, meta-analyses and Mendelian randomization studies. Therefore, the widely implemented concept of high-intensity statin therapy must be replaced by high-intensity LDL-lowering therapy. The efficacy of several drug combinations is supported by scientific evidence and should be considered in the design of lipid-lowering therapies; however, the cost-benefit balance must be taken into account, particularly when prescribing PCSK9 inhibitors. Therapeutic efficiency is a complex issue that varies country to country, and it could be a limitation to full implementation of evidence-based medicine guidelines.

Combination therapies increase efficacy and reduce side effects associated with higher doses, increasing tolerability and leading to higher adherence. A higher efficacy and adherence will result in a higher number of patients achieving recommended therapy targets, which currently is unacceptably low.
